# Molecular Identification and Phylogeny of Natural *Wolbachia* Infection in Iranian Mosquitoes With a New Record of *Wolbachia* Supergroup B Infecting *Anopheles hyrcanus* Populations

**DOI:** 10.1155/jotm/3704339

**Published:** 2026-06-27

**Authors:** Fatemeh Askari, Azim Paksa, Shahin Saeedi, Aioub Sofizadeh, Aboozar Soltani

**Affiliations:** ^1^ Student Research Committee, Department of Medical Entomology and Vector Control, School of Health, Shiraz University of Medical Sciences, Shiraz, Iran, sums.ac.ir; ^2^ Department of Medical Entomology and Vector Control, School of Health, Shiraz University of Medical Sciences, Shiraz, Iran, sums.ac.ir; ^3^ Infectious Diseases Research Center, Golestan University of Medical Sciences, Gorgan, Iran, goums.ac.ir; ^4^ Research Center for Health Sciences, Institute of Health, Department of Medical Entomology and Vector Control, School of Health, Shiraz University of Medical Sciences, Shiraz, Iran, sums.ac.ir

**Keywords:** *Anopheles hyrcanus*, *Culex*, Iran, phylogeny, *Wolbachia*, wsp protein

## Abstract

**Background:**

Mosquitoes are significant vectors of infectious diseases such as malaria and arboviruses, which pose public health challenges globally. *Anopheles hyrcanus* is widely distributed in northern Iran and neighboring Middle Eastern countries and is considered a potential vector of mosquito‐borne diseases in these regions. *Wolbachia*, an endosymbiotic bacterium, is known for its reproductive manipulations and potential to reduce the transmission of mosquito‐borne diseases. This study aimed to investigate the molecular identification and phylogeny of *Wolbachia* in mosquito species from Golestan Province, Iran, with a focus on *Anopheles hyrcanus*, *Culex theileri*, *Culex tritaeniorhynchus*, and *Culex pipiens*.

**Methods:**

Adult mosquitoes were collected from nine locations in Golestan Province between April and December 2023. DNA was extracted from these mosquitoes, and the *Wolbachia* surface protein (WSP) gene was amplified via PCR. The resulting sequences were analyzed to determine phylogenetic relationships using the maximum likelihood method.

**Results:**

A total of 491 female mosquitoes representing six species were morphologically identified, of which five species were screened for *Wolbachia* infection using wsp‐PCR. Infection was detected in four species. The observed infection prevalence among tested specimens was 100% in *Culex pipiens* (15/15; 95% CI: 79.6%–100%), 100% in *Culex theileri* (1/1 pooled sample; 95% CI: 20.6%–99.9%), 37.5% in *Culex tritaeniorhynchus* (15/40; 95% CI: 24.2%–53.0%), and 25% in *Anopheles hyrcanus* (10/40; 95% CI: 14.2%–40.2%), while *Anopheles maculipennis* tested negative (0/9; 95% CI: 0.0%–29.9%). Phylogenetic analysis revealed that all detected strains clustered within *Wolbachia* Supergroup B. Notably, this study represents the first report of *Wolbachia* Supergroup B detected in *Anopheles hyrcanus* in the Middle East region.

**Conclusions:**

The detection of *Wolbachia* Supergroup B in *Anopheles hyrcanus* provides new insights into the distribution and phylogeny of *Wolbachia* in Iranian mosquito populations. However, these findings represent preliminary molecular evidence, and further experimental studies are required to determine infection stability, transmission dynamics, and any potential implications for vector control.

## 1. Introduction

Mosquitoes are of significant medical importance as they are vectors for various pathogens, including viruses and parasites, responsible for diseases such as malaria, dengue fever, Zika virus, and West Nile virus (WNV), posing serious public health challenges worldwide.

Iran is currently in the malaria elimination phase, with indigenous transmission largely restricted to southeastern provinces such as Sistan and Balouchestan and Hormozgan. Golestan Province, located in northern Iran, is not considered an active malaria‐endemic focus; however, it remains epidemiologically relevant due to the presence of competent *Anopheles* vectors and suitable ecological conditions for mosquito breeding. Sporadic imported malaria cases have been reported in nonendemic regions of Iran, highlighting the importance of continued entomological surveillance [1].

In northern Iran, including Golestan Province, several arboviruses of public health importance have been documented, most notably WNV. Seroepidemiological studies have reported WNV exposure rates ranging from approximately 5% to over 20% in human and animal populations in different parts of northern Iran, indicating active or recent transmission cycles. Additionally, WNV RNA has been detected in *Culex* mosquito populations, particularly *Cx. pipiens* and *Cx. tritaeniorhynchus*, which are abundant in this region [2]. Although large‐scale outbreaks are relatively rare, the consistent detection of viral circulation highlights the ecological suitability of northern Iran for arbovirus maintenance.

In southwestern Asia, the Culicidae family comprises seven genera and over 98 species of mosquitoes. The latest checklist of Iranian mosquitoes identifies species from both subfamilies, encompassing a total of 70 species classified within 8–12 genera, contingent upon the classification system applied to the Aedini subfamily [3, 4].

Among the *Anopheles* species reported in Iran, *Anopheles hyrcanus* has been documented in northern regions, including Golestan Province, where ecological conditions support its persistence. However, unlike primary malaria vectors such as *An. stephensi* and members of the *An. maculipennis* complex, *An. hyrcanus* is generally considered a secondary or potential vector with a limited or context‐dependent role in malaria transmission [4–6]. Its epidemiological importance in northern Iran remains uncertain, particularly in areas where malaria transmission has been reduced or interrupted. Nevertheless, its widespread distribution and ecological adaptability justify continued investigation, particularly in studies exploring mosquito‐associated microbiota.

*Wolbachia* (order Rickettsiales) is a maternally transmitted intracellular endosymbiotic bacterium estimated to infect approximately 40% of arthropod species [7]. It is best known for manipulating host reproduction through mechanisms such as cytoplasmic incompatibility (CI), parthenogenesis, feminization, and male killing, thereby enhancing its own transmission within host populations [8]. Currently, *Wolbachia* strains are classified into at least 17 supergroups (A–R), with supergroups A and B being the most commonly reported in mosquitoes. Despite increasing global interest, the infection status of the majority of culicid species remains poorly characterized, particularly in understudied geographic regions. This knowledge gap highlights the importance of region‐specific molecular surveys to better understand the diversity, distribution, and potential epidemiological relevance of *Wolbachia* in mosquito vectors [9].

Among the reproductive alterations induced by *Wolbachia* is CI, a phenomenon that affects reproductive success in infected hosts [7]. In addition to its impact on reproduction, the presence of *Wolbachia* within mosquito species can significantly affect their vector competence. Specifically, certain *Wolbachia* strains have demonstrated the ability to decrease the viral load of specific pathogenic viruses within mosquitoes, thus potentially limiting the transmission of these viruses to humans and other animals [10]. This dual role of *Wolbachia*—altering host reproductive dynamics while influencing virus transmission—illustrates its complex interactions in the host ecosystem and presents avenues for further research into biological control measures for vector‐borne diseases.

Natural infection of mosquitoes by *Wolbachia* strains can inhibit the transmission of human arboviruses within mosquito populations, thereby playing a crucial role in public health. As a result, there is growing interest in harnessing *Wolbachia* for biocontrol strategies designed to reduce the transmission of mosquito‐borne diseases. This momentum has been bolstered by the discovery of natural *Wolbachia* strains in various vector species, particularly in tropical and subtropical regions, where environmental conditions favor their establishment [11].

However, the prevalence and density of these *Wolbachia* strains are not uniform across different mosquito species, varying significantly based on geographical location and climate. Identification of novel natural *Wolbachia* strains suggests that their prevalence and diversity may have been substantially underestimated in previous studies. This discrepancy underscores the need for extensive surveys and genetic analyses to more accurately assess the distribution of these strains.

In this context, our study aims to conduct a survey of *Wolbachia* strains in mosquitoes through molecular assays in a lesser‐studied region of northern Iran (Golestan province). This region will provide a unique opportunity to explore the diversity and phylogenetic relationships of *Wolbachia* present in local mosquito populations. By comparing these strains with their counterparts from other regions of Iran and across the globe, this research seeks to contribute valuable insights into the ecological roles of *Wolbachia* and its potential applications in biocontrol strategies aimed at mitigating the burden of mosquito‐borne diseases. Ultimately, a comprehensive understanding of *Wolbachia*’s distribution and function will enhance our capacity to develop effective public health interventions.

## 2. Material and Methods

### 2.1. Study Area

Golestan province, located in northeastern Iran along the Caspian Sea, spans approximately 20,437.74 km^2^ and is divided into 11 districts, housing a population of about 1.6 million residents. The region’s varied landscape results in three primary climate types: moderate plains, mountainous areas, and semi‐arid zones. According to data obtained from the Iran Meteorological Organization (IMO), based on 10‐year climatological averages (2013–2023), Golestan Province has a mean annual rainfall of approximately 556 mm and an average annual temperature of 18.2°C.

**FIGURE 1:**
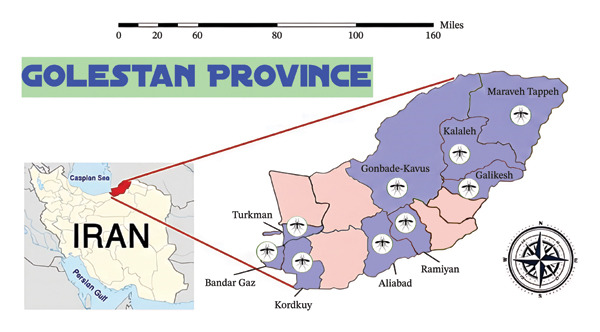
GIS‐based map showing the geographic distribution of nine sampled counties in Golestan Province, northeastern Iran (2023). Exact coordinates are provided in Table 1.

From April to December 2023, mosquito sampling was conducted in nine counties across Golestan Province: Ramiyan, Kordkuy, Aliabad, Gonbade‐Kavus, Bandar Gaz, Turkman, Galikesh, Maraveh Tappeh, and Kalaleh (Figure 1; Table 1). Within each county, three distinct microhabitat sampling points were selected to capture local ecological variability, resulting in a total of 27 collection points across the province. The selection of counties was based on representation of the province’s three principal climatic zones (moderate plains, mountainous areas, and semi‐arid regions) to ensure geographic and environmental diversity in mosquito collection. Sampling took place in the southern areas (specifically Ramiyan and Aliabad), the northern region (Gonbade‐Kavus), the western districts (including Kordkuy, Bandar Gaz, and Turkman), and the eastern sites (namely, Galikesh, Maraveh Tappeh, and Kalaleh) (Table 1).

**TABLE 1:** Geographic characteristics of study areas in Golestan Province, northeastern Iran, April–December 2023.

NO.	Locations	Latitude (*N*)	Longitude (E)	Altitude (M)
1	Ramiyan	37.01,468,500°N	55.14,038,900°E	226
2	Kordkuy	36.7917°N	54.1133°E	50
3	Aliabad	36.941,857°N	54.571,879°E	140
4	Gonbade‐Kavus	37.15°N	55°E	52
5	Bandar Gaz	36.7775°N	53.9489°E	−14
6	Turkman	36.909,722°N	54.113,611°E	−20
7	Galikesh	37.2661°N	55.4367°E	210
8	Maraveh Tappeh	37.90,430,400°N	55.95,454,000°E	206
9	Kalaleh	37.38,207,145°N	55.49,228,362°E	160

A high‐resolution geographic information system (GIS)–based map was generated to accurately illustrate the spatial distribution of sampling locations within Golestan Province (Figure 1). Geographic coordinates for each county were recorded using handheld GPS devices during field collection and verified using official administrative boundary datasets. Spatial visualization and map preparation were performed using ArcGIS software version 10.8 (Esri, Redlands, CA, USA).

### 2.2. Sampling Methods for Adult Mosquitoes

Adult mosquitoes were collected using a variety of methodologies, including hand capture, both night and day landing collections, pyrethrum spray catch techniques, and UV light trap sampling. The hand capture method utilized manual aspirators for a duration of approximately 20 min, both outdoors and indoors. UV light traps were operated biweekly, while landing catches involved the use of human and animal baits. The collected specimens were subsequently transported to the entomology laboratory for identification, during which comprehensive data regarding the geographic, climatic, and environmental attributes of the sampling locations were documented [3]. Specimens were identified morphologically using Harbach and Azari‐Hamidian & Harbach taxonomic keys [5]. Morphology was chosen as the primary identification method because it permitted rapid, field‐level species assignment and population counts across the nine sampling sites. They were stored in a freezer at −20 degrees Celsius until molecular investigations.

### 2.3. Molecular Study

DNA was extracted from whole adult females following Collins et al. [12]. We recognize that whole‐body extraction increases the likelihood of detecting *Wolbachia* DNA from nongermline sources (gut contents, ectoparasites/parasitoids, or environmental contamination). Therefore, detection in whole bodies should be interpreted as evidence of presence rather than definitive proof of stable, maternally transmitted infection. Confirmatory approaches (recommended for future work) include dissection and separate extraction of ovaries/germline tissues, surface sterilization prior to DNA extraction, and *Wolbachia* density estimation by qPCR, which together can distinguish true endosymbiosis from transient or environmental DNA signals.

The *Wolbachia* surface protein (WSP) genes were amplified via polymerase chain reaction (PCR) to explore the presence and genetic diversity of *Wolbachia* strains in *An. hyrcanus, Cx. tritaeniorhychus Cx. theileri* and *Cx. pipiens* mosquitoes. WSP was selected as an initial screening marker because it is sensitive for *Wolbachia* detection and widely used in field surveys [13]. The primer specifications and PCR thermal cycles are detailed below [13, 14]:

The Maxime PCR Premix Kit (i‐Taq), Cat. No. 25025 from INtRON Company, was used for the reactions. Each PCR step included 2.5 μL of forward and reverse primers and 5 μL of DNA in the initial reaction. Each PCR run included a positive control (*Drosophila Wolbachia* DNA) and a negative water control. To minimize contamination, we performed extractions and PCR setup in separate rooms, used filter tips, and included extraction blanks. PCR products were visualized on 1% agarose gels (Tables 2 and 3). Each sequence was compared with those in GenBank using the NCBI database.

**TABLE 2:** The thermal program for PCR amplification of the wsp gene related to *Wolbachia* infection in adult mosquitoes.

Step	Temperature (degrees Celsius)	Time (minute)	Number of cycles
Initial denaturation	95	5	1
Denaturation	94	1	35
Annealing	55	1	
Extension	72	1	
Final extension	72	7	1

**TABLE 3:** Specifications of primers used in this study for PCR and sequencing of the wsp gene.

Primer	Primer sequence	Function	Primer length (bp)	Melting temperature (C)
81F	TGG TCC AAT AAG TGA TGA AGA AAC	Forward	24	66
691R	AAA AAT TAA ACG CTA CTC CA	Reverse	20	52

The obtained wsp sequences were initially edited and trimmed to remove low‐quality regions using BioEdit v7.2.5. Multiple sequence alignment was performed using the ClustalW algorithm implemented in MEGA version 6.0. Alignment accuracy was visually inspected and manually adjusted where necessary to ensure positional homology [15, 16]. Reference wsp sequences representing established *Wolbachia* supergroups were retrieved from GenBank to provide comparative context.

Phylogenetic relationships were inferred using the maximum likelihood (ML) method implemented in MEGA 6.0. The best‐fitting nucleotide substitution model was determined based on the lowest Bayesian Information Criterion (BIC) score, and the Kimura 2‐parameter (K2P) model was selected for tree construction. Statistical support for branching patterns was evaluated using 1000 bootstrap replicates. Only bootstrap values ≥ 70% were considered indicative of strong node support.

Representative sequences from *Wolbachia* supergroup C were included as an outgroup to root the phylogenetic tree and improve supergroup resolution. The final tree was visualized and edited for publication clarity. All newly generated sequences were deposited in GenBank.

The detection of *Wolbachia* DNA using the wsp marker should be interpreted as molecular evidence of bacterial presence rather than definitive confirmation of stable, vertically transmitted endosymbiosis. The wsp gene is widely used for screening due to its high sensitivity; however, it is also known to exhibit recombination and elevated evolutionary rates, which may limit its phylogenetic robustness. Furthermore, whole‐body DNA extraction increases the possibility of detecting environmental DNA, transient infections, or DNA originating from gut contents or parasitoids. Although strict contamination controls were implemented (separate extraction rooms, filter tips, extraction blanks, positive and negative controls), confirmatory approaches such as MLST typing (gatB, coxA, hcpA, fbpA, ftsZ), quantitative PCR for density estimation, fluorescence in situ hybridization (FISH), and tissue‐specific dissections targeting ovaries would be necessary to verify stable maternal transmission and intracellular localization. Therefore, the present findings should be considered an initial molecular survey rather than definitive strain characterization [9, 17–19].

### 2.4. Statistical Analysis

Prevalence estimates were calculated as proportions, and 95% confidence intervals (CIs) were computed using the Wilson score method, which is appropriate for binomial data, particularly in cases of small sample sizes or extreme proportions.

### 2.5. Results

A total of 819 adult mosquitoes were collected from nine counties in Golestan Province, Iran, between April and December 2023. Of these, 491 female mosquitoes were identified morphologically using validated taxonomic keys. Six species were identified: four culicine and two anopheline. The most abundant was *Cx. tritaeniorhynchus*, comprising 84.93% of the total, followed by *An. hyrcanus* at 8.55%. Other species included *Cx. pipiens* (3.46%), *An. maculipennis* (1.83%), and *Cx. theileri* (1.02%). *Cx. territans* had the lowest frequency at 0.20%.

**FIGURE 2:**
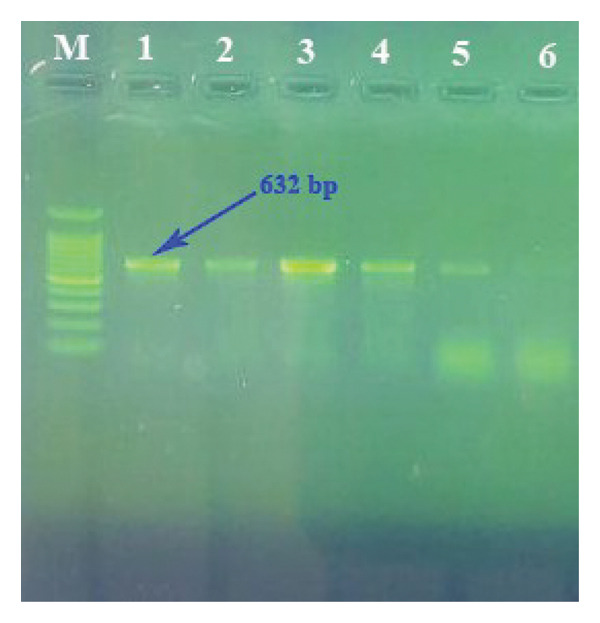
Electrophoresis of the PCR for a part of wsp gene of 632 bp length in different populations of mosquitoes collected from different parts of Golestan Province, Iran (M marker 100 bp, 1: *Drosophila melanogaster* (positive control), 2–5: *Culex theileri*, *Culex tritaeniorhychus*, *Anopheles hyrcanus*, *Culex pipiens*, 6: negative control).

All species except *Cx. territans*, which had only one adult captured, underwent molecular analysis for *Wolbachia* detection. Among the five species analyzed, all except *An. maculipennis* tested positive for *Wolbachia* bacteria (Figure 2). The four positive species, which were collected from different locations, were further analyzed and sequenced. Table 4 reports the morphologically identified individuals, those tested by wsp‐PCR, the PCR‐positive counts and percentages, and the GenBank sequence numbers (Table 4).

**TABLE 4:** Detection of *Wolbachia* infection in mosquito species collected from northeastern Iran.

Species	Number identified (morphology)	Number tested by PCR	Number positive by PCR	% positive (of tested)	95% CI (Wilson)	Number sequenced (GenBank accession numbers)
Cx. tritaeniorhynchus	417	40	15 (3 pools of 5 mosquito bodies)	37.5%	24.2%–53.0%	3 (PQ192628–PQ192630)
An. hyrcanus	42	40	10 (2 pools of 5 mosquito bodies)	25%	14.2%–40.2%	2 (PQ192631–PQ192632)
Cx. pipiens	17	15	15 (3 pools for 5 mosquito bodies)	100%	79.6%–100%	3 (PQ192633–PQ192635)
An. maculipennis	9	9	0	0	0.0%–29.9%	—
Cx. theileri	5	5	1 (1pools of 5 mosquito bodies)	100%	20.6%–99.9%	1 (PQ192627)
Cx. territans	1	1	0	0	0.0%–79.4%	—

*Note:* 1. Cx. = *Culex*; *An.* = *Anopheles.* 2. “Number tested by PCR” refers to female adults screened by wsp‐specific PCR assay. 3. “% positive” calculated as (number positive/number tested) × 100. 4. GenBank accession numbers correspond to confirmed positive samples sequenced in this study.

Due to relatively small and uneven sample sizes across species, particularly for *Culex theileri*, *Culex pipiens*, and *Culex territans*, the study was not statistically powered to support formal interspecific comparisons of *Wolbachia* prevalence.

### 2.6. Phylogenetic Relationship

**FIGURE 3:**
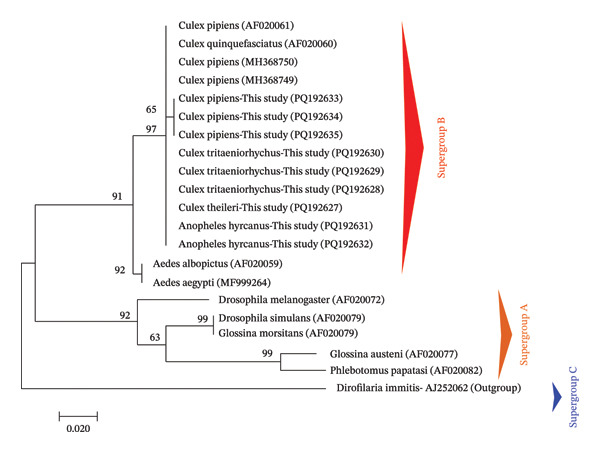
Molecular phylogenetic analysis using the maximum likelihood method based on the wsp gene for samples of *Culex theileri*, *Culex tritaeniorhychus*, *Culex pipiens*, and *Anopheles hyrcanus* collected in Golestan Province, Iran. The *Wolbachia* sequences showed a similarity of 99%–100%. Supergroup B was identified, with Supergroup C used as an outgroup.

*Wolbachia* sequences were obtained from infected individuals of *Culex theileri*, *Culex tritaeniorhychus*, *Culex pipiens*, and *Anopheles hyrcanus* in this study. The final alignment consisted of approximately 600 bp after trimming ambiguous ends. Each sequence was registered in GenBank (Table 5). Molecular phylogenetic analysis using the ML method based on the wsp gene indicated that *An. hyrcanus*, *Cx. theileri*, *Cx. pipiens*, and *Cx. tritaeniorhychus* specimens were infected with strains of the B *Wolbachia* supergroup. The *Wolbachia* wsp sequences exhibited high similarity (99%–100%), indicating that most sequences were near‐identical with minor nucleotide differences, although a small number of sequences were completely identical across samples after alignment trimming (Figure 3).

**TABLE 5:** GenBank accession numbers of *Wolbachia* sequences in infected mosquito species from Golestan Province, Iran, 2024.

Sampling locations	Species	Accession numbers
Galikesh	*Culex theileri*	PQ192627
Ramiyan	*Culex tritaeniorhychus*	PQ192628
Kalaleh	*Culex tritaeniorhychus*	PQ192629
Galikesh	*Culex tritaeniorhychus*	PQ192630
Turkman	*Anopheles hyrcanus*	PQ192631
Turkman	*Anopheles hyrcanus*	PQ192632
Ramiyan	*Culex pipiens*	PQ192633
Aliabad	*Culex pipiens*	PQ192634
Kalaleh	*Culex pipiens*	PQ192635

## 3. Discussion

This study provides comprehensive molecular identification and phylogenetic analysis of *Wolbachia* infection in mosquito populations from Golestan Province, Iran, with a focus on four mosquito species: *Culex theileri*, *Culex tritaeniorhynchus*, *Culex pipiens*, and *Anopheles hyrcanus*. The detection of *Wolbachia* across multiple species in this region highlights the widespread nature of this endosymbiotic bacterium in Iranian mosquito populations and aligns with global findings on *Wolbachia*’s adaptability to diverse mosquito species and geographical areas [7, 8]. This study adds to the growing body of research, suggesting that *Wolbachia* could play an important role in mosquito population dynamics and the transmission of mosquito‐borne diseases, particularly in regions like Iran where arboviral and parasitic infections are prevalent.

A closer examination of the quantitative results reveals notable interspecific differences in *Wolbachia* detection rates. Among the species screened, *Culex pipiens* exhibited the highest prevalence (100% of tested pools), followed by *Culex theileri* (100% in the single tested pool), whereas *Culex tritaeniorhynchus* showed moderate prevalence (37.5%) and *Anopheles hyrcanus* demonstrated comparatively lower detection (25%). In contrast, *Anopheles maculipennis* was negative in all tested specimens. These interspecific differences may reflect ecological variation in breeding habitats, host–microbiome interactions, vertical transmission efficiency, or historical exposure to *Wolbachia*. The high prevalence observed in *Culex pipiens* aligns with global reports indicating stable and widespread *Wolbachia* infections in this species complex. Conversely, the lower detection rate in *Anopheles hyrcanus* may indicate either lower infection stability, reduced bacterial density, or geographically restricted distribution within Golestan Province. Site‐level variation further suggests that environmental factors such as temperature, humidity, larval habitat type, and population structure could influence infection persistence. These findings underscore the importance of species‐specific and region‐specific evaluation when assessing *Wolbachia* epidemiology.

Despite these findings, the presence of *Wolbachia* in *Anopheles* mosquitoes remains a subject of active scientific debate. Several studies have reported natural infections in field populations, while others argue that low‐density detection may reflect environmental contamination, transient acquisition, or amplification artifacts rather than stable endosymbiosis. The inconsistent detection of *Wolbachia* in *Anopheles* across geographic regions, combined with variability in infection density and transmission patterns, underscores the need for cautious interpretation. Therefore, while our molecular results support the presence of *Wolbachia* DNA in *Anopheles hyrcanus*, further validation is required to confirm its biological stability and epidemiological significance [20–22].

A key finding of this study is the identification of *Wolbachia* Supergroup B in *Anopheles hyrcanus* populations, marking the first report of this supergroup in this species in the Middle East region. This is notable because *Anopheles* mosquitoes have historically been underrepresented in *Wolbachia* research compared to *Aedes* and *Culex* species. Previous studies have reported variable and often low‐density *Wolbachia* infections in *Anopheles*, with inconsistent detection across geographic regions. The clustering of all sequences within Supergroup B is consistent with global observations of *Wolbachia* diversity in mosquitoes and suggests a relatively conserved phylogenetic pattern among the strains detected in this study [7, 9, 14, 23–27].

Although the detection of *Wolbachia* Supergroup B in *Anopheles hyrcanus* represents a novel regional finding, its implications for malaria transmission remain uncertain. Experimental studies in other *Anopheles* species have produced variable results, with some demonstrating reduced *Plasmodium* development and others reporting neutral or context‐dependent effects. The impact of *Wolbachia* on vector competence appears to depend on strain type, bacterial density, host genotype, and environmental conditions. Therefore, while the presence of *Wolbachia* in *Anopheles hyrcanus* raises intriguing possibilities for biological control, the effects of this strain on *Plasmodium* transmission dynamics and mosquito population structure require experimental validation. Controlled laboratory assays assessing parasite inhibition, vertical transmission efficiency, and infection density will be essential before drawing conclusions regarding epidemiological significance [10, 11, 20, 21].

However, the biological implications of detecting Supergroup B in *Anopheles hyrcanus* must be interpreted cautiously. Unlike *Aedes* and *Culex*, *Anopheles* species have historically been considered refractory or only sporadically infected by *Wolbachia*. Some investigators propose that stable infections in *Anopheles* are rare and may require specific ecological or microbiome contexts to persist. Others suggest that naturally occurring infections may be low density and insufficient to induce classical reproductive phenotypes such as CI. Additionally, recent meta‐analyses emphasize that wsp‐based detection alone cannot distinguish between stable intracellular infections and extracellular DNA presence [9]. Therefore, while our findings expand the documented distribution of Supergroup B, functional studies examining vertical transmission rates, bacterial density, reproductive effects, and impact on *Plasmodium* development are essential before drawing conclusions regarding vector control applications.

Wong et al. reported the first natural infection of *Wolbachia* in field‐collected *An. hyrcanus* in Malaysia. Their research revealed a 50% infection rate, with 9 out of 18 specimens testing positive for *Wolbachia*, highlighting its presence in a species previously believed to be free of this endosymbiotic bacterium [28]. This discovery contrasts with earlier studies, such as that by Baldini et al., which found lower infection rates in *An. gambiae* populations in Africa, suggesting geographic and species variation in *Wolbachia* presence [20]. The discovery of stable *Wolbachia* infections in *Anopheles hyrcanus* opens new avenues for research, particularly in exploring how *Wolbachia* infections could influence malaria transmission dynamics in regions where *Anopheles* species are key malaria vectors.

Although *Wolbachia*‐based strategies have shown promise in reducing pathogen transmission in some mosquito species [10], particularly *Aedes*, the implications of detecting *Wolbachia* in *Anopheles hyrcanus* remain uncertain [29]. The present study provides molecular evidence of *Wolbachia* DNA but does not assess bacterial density, tissue localization, vertical transmission, or effects on *Plasmodium* development. Consequently, no conclusions can be drawn regarding its role in malaria transmission or suitability for control interventions. The potential application of *Wolbachia* in *Anopheles* mosquitoes requires rigorous validation through controlled laboratory experiments and field‐based studies evaluating infection stability, host fitness effects, and parasite interference.

Moreover, the high level of similarity (99%–100%) among the *Wolbachia* strains detected in different mosquito species suggests that these bacteria may be more widespread and uniform across geographical regions than previously thought [30]. This level of similarity reflects near‐identical sequences rather than complete uniformity, suggesting limited genetic variation within the sampled populations. This finding is consistent with global studies that report similar genetic relationships among *Wolbachia* strains in mosquito populations, suggesting that horizontal gene transfer or cross‐species transmission might play a significant role in maintaining *Wolbachia* populations across diverse environments [13]. This genetic similarity, particularly within Supergroup B, is of considerable interest because Supergroup B *Wolbachia* has been associated with reproductive manipulations such as CI, parthenogenesis, and male‐killing [7]. These effects can significantly alter mosquito population dynamics, leading to population suppression or replacement, both of which are crucial for controlling vector‐borne diseases.

The detection of *Wolbachia* in *Culex pipiens* and *Culex tritaeniorhynchus* is not surprising given the high prevalence of *Wolbachia* in these species reported worldwide [30]. *Culex* mosquitoes are important vectors for arboviruses such as WNV, and *Wolbachia* infections in these species could reduce their vector competence, as seen in other studies [9, 31]. This is particularly relevant in northern Iran, where WNV circulation has been documented, with seroprevalence estimates in some studies exceeding 10% in exposed populations and viral detection reported in *Culex* vectors. The widespread infection of *Culex* species with *Wolbachia* offers significant opportunities for vector control programs targeting arboviral diseases, which are prevalent in both urban and rural areas of Iran [32]. The ability of *Wolbachia* to inhibit arbovirus replication within mosquitoes has been well documented, and this property could be harnessed to reduce the transmission of diseases like West Nile fever and Japanese encephalitis in the region [33].

The molecular phylogenetic analysis of the *Wolbachia* strains infecting mosquitoes in this study revealed a close relationship between the strains, with all falling within Supergroup B. This finding suggests that the *Wolbachia* strains infecting these mosquito species are either the same or closely related strains [30, 34]. The use of Supergroup C as an outgroup in this analysis further strengthens the phylogenetic classification and suggests that the *Wolbachia* strains detected in this study are likely to have similar evolutionary origins [13]. This raises important questions about the mechanisms driving the persistence and spread of *Wolbachia* across different mosquito species in Iran and other regions. Horizontal transmission between species, along with vertical transmission within species, may both contribute to the high prevalence and genetic uniformity observed in this study [7]. Importantly, phylogenetic placement within Supergroup B alone does not imply functional effects such as CI or pathogen blocking, and such phenotypes cannot be inferred from wsp‐based sequence data.

However, while the findings of this study are promising, there are still many unknowns regarding the role of *Wolbachia* in *Anopheles* mosquitoes and the potential for these infections to affect malaria transmission. The relationship between *Wolbachia* and *Plasmodium* is complex and not yet fully understood. Although some studies have suggested that *Wolbachia* infections could reduce the transmission of *Plasmodium* by *Anopheles* mosquitoes, the effects are not always consistent, and more research is needed to clarify the mechanisms involved [21]. Future studies should focus on investigating whether *Wolbachia* infections in *Anopheles hyrcanus* affect their susceptibility to *Plasmodium* infection and their capacity to transmit malaria. Such research could have profound implications for malaria control efforts in Iran and other endemic regions.

The findings from this study also highlight the importance of expanding *Wolbachia* research beyond the traditional focus on culicine mosquitoes. *Anopheles* species, particularly in regions where malaria is endemic, may harbor *Wolbachia* infections that could be exploited for biocontrol purposes. Given the increasing resistance of malaria vectors to insecticides, alternative strategies, such as *Wolbachia*‐based interventions, are urgently needed to reduce the burden of malaria. Additionally, future research should explore the interactions between *Wolbachia* and other endosymbionts, such as microsporidia, which may coinfect mosquitoes and further influence disease transmission dynamics [31].

Limitations of this study include (i) morphological identification without molecular confirmation for cryptic species complexes (e.g., members of the *An. maculipennis* complex). For definitive species assignment in future surveys, we recommend supplementing morphological identification with molecular barcoding (COI) or species‐complex‐specific PCR assays, (ii) whole‐body DNA extraction, which can detect environmental or nongermline *Wolbachia* DNA, and (iii) reliance on wsp for phylogenetic inference. These are partially offset by rigorous field sampling, sequencing of positive products, and GenBank deposition, but follow‐up MLST, tissue‐specific sampling, and COI barcoding are needed to confirm these preliminary findings. In addition, the limited and imbalanced sample sizes across mosquito species restricted the statistical power for comparative analyses of *Wolbachia* prevalence between species.

WSP was selected as an initial screening marker because it is sensitive for *Wolbachia* detection and widely used in field surveys. However, we explicitly treat these data as exploratory (screening) because the wsp gene is known to be highly variable and subject to recombination, which can confound deeper phylogenetic inference. For definitive strain typing and robust phylogenetic placement, we recommend MLST (gatB, coxA, hcpA, fbpA, ftsZ) and 16 S rRNA/groEL sequencing and concatenated analyses.

## 4. Conclusions

This study provides a regional molecular survey of *Wolbachia* infection in mosquito species from northern Iran and reports, for the first time, the presence of *Wolbachia* Supergroup B in *Anopheles hyrcanus* in the Middle East. These findings expand current knowledge on *Wolbachia* distribution and genetic diversity in understudied vector populations. However, the results should be interpreted as preliminary, as detection was based on wsp‐PCR from whole‐body extracts and does not confirm stable, maternally transmitted endosymbiosis. No inferences can be made regarding effects on mosquito biology, vector competence, or malaria transmission. Future studies employing multilocus sequence typing (MLST), tissue‐specific assays, and experimental infection models are necessary to validate these findings and determine their biological and epidemiological significance. By expanding our understanding of *Wolbachia* in mosquito populations, we can develop innovative and sustainable strategies for controlling mosquito‐borne diseases in Iran and beyond.

## Author Contributions

Aboozar Soltani designed and conceptualized the study. Fatemeh Askari, Shahin Saeedi, and Aioub Sofizadeh gathered the data. Aboozar Soltani, Azim Paksa, and Shahin Saeedi analyzed them. Fatemeh Askari and Aboozar Soltani drafted the manuscript. All the authors participated in writing the manuscript.

## Funding

This research was supported by the Vice‐chancellor for Research and Technology Affairs of the Shiraz University of Medical Sciences (grant number: 29437), Shiraz, Iran.

## Disclosure

All the authors have read and approved the final manuscript.

## Ethics Statement

All study procedures were conducted in compliance with the Declaration of Helsinki and were approved by the Iran National Committee for Ethics in Biomedical Research (Approval ID: IR.SUMS.REC.1402.555).

## Conflicts of Interest

The authors declare no conflicts of interest.

## Data Availability

The datasets generated and/or analyzed during the current study are available in the GenBank repository [accession numbers: PQ192627‐35].
